# Cultural Scripts of Traumatic Stress: Outline, Illustrations, and Research Opportunities

**DOI:** 10.3389/fpsyg.2019.02528

**Published:** 2019-11-15

**Authors:** Yulia Chentsova-Dutton, Andreas Maercker

**Affiliations:** ^1^Department of Psychology, Georgetown University, Washington, DC, United States; ^2^Department of Psychology, University of Zurich, Zurich, Switzerland; ^3^Institute of Advanced Study Berlin, Berlin, Germany

**Keywords:** cultural scripts, posttraumatic stress disorder, traumatic stress, illness narratives, cultural clinical psychology

## Abstract

As clinical-psychological scientists and practitioners increasingly work with diverse populations of traumatized people, it becomes increasingly important to attend to cultural models that influence the ways in which people understand and describe their responses to trauma. This paper focuses on potential uses of the concept of cultural script in this domain. Originally described by cognitive psychologists in the 1980s, scripts refer to specific behavioral and experiential sequences of elements such as thoughts, memories, attention patterns, bodily sensations, sleep abnormalities, emotions and affective expressions, motivation, coping attempts, and ritualized behaviors that are relevant to posttraumatic adjustment. We differentiate between experiences of traumatic stress that are scripted (e.g., cultural explanations are available) versus unscripted. Further characteristics such as script tracks, the effect of script interruptions, and contextual fit of scripts with other cultural models are also described. We consider examples of traumatic stress associated with war and organized, sexualized violence from “Western” and “non-Western” world regions. The concluding part of this review describes a number of possibilities for methodological approaches to assessment of cultural scripts. Capturing central elements of the script(s) of trauma would aid psychological researchers and clinicians in understanding the experiences of trauma in cultural context, which could ultimately lead to better clinical service opportunities worldwide.

Culture matters when it comes to understanding and treating psychological sequelae of traumatic events (Marsella, 2010). Cultural contexts differ in the current and historical risks of exposure to many forms of trauma (Benjet et al., 2016). Shaped in part by local adaptations to these community-specific risks, different beliefs about posttraumatic adjustment emerge in different contexts (De Jong, 2004; Hinton and Lewis-Fernández, 2010; Nader et al., 2013; Maercker et al., 2019). When faced with broadly similar traumatic events, such as wars or mass violence, people from different cultural contexts foreground different aspects of traumatic stress, such as nightmares, numbing, or being haunted by ghosts in their descriptions of distress (De Jong et al., 2001; Norris et al., 2001a). Evidence also suggests that prevalence rates of Posttraumatic Stress Disorder (PTSD) and symptoms of traumatic stress depend in part on cultural factors (Perilla et al., 2002; Jobson and O’Kearney, 2009; Burri and Maercker, 2014). How do we make sense of cultural influences in this domain? Prior work suggests that traumatic stress can be understood not just as a set of objective circumstances and physiological and psychological responses to them, but as shared cultural understandings that affect these responses (Meštrović, 1985), a broad set of concept known as *cultural models* (d’Andrade and Strauss, 1992) and *mental models* (Jones et al., 2011).

In this review, we will introduce literature on cultural models to a psychological audience with a goal of fostering more work on this topic. We will rely on a more specific term, *cultural scripts* (or subtypes of cultural models encoding sequenced sets of specific changes), to highlight the fact that many models in this domain are dynamic, describing ways in which symptoms are thought to evolve over time and respond to available treatments and coping strategies. We will draw on work in anthropology, transcultural psychiatry, and cultural and clinical psychology to: (1) introduce the concept of cultural scripts; (2) apply it to cultural models of responses to trauma; and (3) describe the potential impacts of cultural scripts on psychological outcomes. We will also consider ways in which this concept can be useful to clinical scientists and practitioners, describing some examples of cultural scripts of posttraumatic distress and offering a review of methodological tools that can be used to assess scripts of trauma in clinical research and practice.

## Complex Phenomenological Fields of Responses to Trauma

Responses to trauma are inherently complex, even confusing, going far beyond the core symptoms described in the major classification systems for mental disorders (Stein et al., 2018). They can involve numerous changes in thoughts, memories, attention, sleep, emotions, motivation, and behavior. Encounters with traumatic events can also affect existential beliefs and spiritual experiences, challenging the ways in which the self is understood. Many traumatic events involve bodily trauma, which has its own medically explained and unexplained sequelae (Roelofs and Spinhoven, 2007; Hinton and Good, 2016). This picture can become further complicated by the fact that posttraumatic distress is not experienced in a social vacuum (Harvey, 1996; Kawachi and Subramanian, 2006). Others may be traumatized as well, and changes in one person may reverberate through the social network (Jeon et al., 2005; Maercker and Horn, 2013). Efforts to regulate expression of distress can change its manifestations (Somasundaram, 2007). Particularly relevant for the concept of cultural scripts, the complex patterns of changes that are triggered by trauma evolve over time (McFarlane and Yehuda, 2000).

Taken together, all changes that are perceptible to a person and close others following a traumatic event, whether or not attributed to trauma, constitute a phenomenological field of responses to trauma. Typically, the sheer number of changes outstrips any succinct definition, making it challenging to track, understand, and describe what is happening. The phenomenological field is also difficult to study, requiring broad and systematic bottom-up assessment of all potential signs of distress (or all psychological or physiological changes that are noticed by the person in the wake of trauma). Not surprisingly, we know little about whether broader phenomenological fields differ across cultural contexts. One study of Mexican trauma survivors suggested that their reported symptoms were indeed much more complex than a typical diagnostic set of symptoms (Norris et al., 2001b). Research on other forms of distress, such as depression, indicates that phenomenological fields differ even across relatively similar cultural contexts (Saint Arnault and Kim, 2008).

## Cultural Models Function to Reduce the Complexity

In contrast to broader sets of changes within the phenomenological field of a given traumatized individual, descriptions of traumatic stress that appear in the clinical literature are far more succinct (e.g., Terr, 1988; Horowitz, 1997; Somasundaram, 2007; Hinton and Lewis-Fernández, 2010), comprising symptoms that are filtered down from the full phenomenological fields by patients and/or clinicians. Hinton et al. (2018) described this discrepancy as a potential “category truncation,” pointing to the need to assess culturally specific sets of symptoms that are representative of local phenomenological fields. Given the complexity of trauma-related changes, it is not surprising that patients filter their own experiences based on a number of considerations, such as what symptoms are attributed to trauma, what hurts the most, what is considered the most problematic or most meaningful, the most appropriate to talk about, and the most likely to elicit support from others. Not everything can be acknowledged. Cultural prohibitions on some topics, such as political oppression, can create additional filters. Consider a Balinese patient studied by Lemelson and Tucker (2017), who was able to openly link his long-standing symptoms to the trauma of witnessing his father’s murder during a state-sponsored massacre only after the fall of the oppressive Suharto regime. Clinicians or therapists of any origin bring additional layers of filtering. They also decide what is important to attend to, with an emphasis on what is common across patients and distinctive to a clinician’s conceptualization of a disorder (e.g., Pearlman and Saakvitne, 1995).

Each of these filters is in part a cultural one. They are applied in accordance with what is culturally meaningful, yielding culturally patterned signatures of illness and wellness (Jayawickreme et al., 2013). Although patients and clinicians attend to and report information anew, they are guided by cultural models of what is painful, important, and desirable that vary across cultures (Kirmayer, 2015). So too do the models of what is normal and functional (e.g., models of emotions and relationships: Eid and Diener, 2001; Campos, 2015). This means that different cultural contexts foster attention to partially overlapping but also distinct sets of posttraumatic symptoms (de Girolamo and McFarlane, 1996). For example, some cultural contexts, such as Native American communities and Cambodia, foster beliefs about spiritual importance of nightmares and their significance for physical and spiritual security as well as for the spiritual paths of diseased relatives. Traumatized individuals in these contexts often report experiencing troubling nightmares (Hinton et al., 2009, 2013; Shore et al., 2009). Nonetheless, such differences emerge against a background of similarities (de Girolamo and McFarlane, 1996): there is no doubt that the cultural filtering is constrained by common biological, psychological, and spiritual signatures of human responses to trauma. As clinical scientists and practitioners increasingly work with diverse populations of traumatized people, it becomes imperative to attend to the cultural filters that shape the ways in which people reduce the phenomenological complexity of their responses to trauma. Extant work, primarily in anthropology and transcultural psychiatry, has developed a rich set of concepts that capture this complexity.

## Conceptual Domain of Cultural Models of Trauma

Given the high prevalence of traumatic experiences like violence or catastrophes, many cultural contexts transmit at least some information about their psychological, spiritual, and social impacts, whether in the form of formal or informal diagnostic categories, stories, or metaphors (e.g., Birmes et al., 2003; Ben-Ezra, 2004). These models of trauma offer comprehensible ways to understand otherwise complex and confusing responses and infuse experienced changes with meanings. Prior work in anthropology (e.g., Hinton and Good, 2016) and transcultural psychiatry (e.g., Hinton and Lewis-Fernández, 2010; Kirmayer, 2015) has developed a number of approaches to describing cultural models of mental illness more broadly and traumatic stress in particular. The most important ones are *illness narratives* (or stories of developing symptoms of mental illness), *idioms of distress* (or culturally patterned ways of expressing distress and responding to such expressions), and *commonsense* or *explanatory models of distress* and *illness or symptom schema* (or appraisals and ideas about causes and nature of particular types of distress; Kleinman, 1977; Meyer et al., 1985; Robbins and Kirmayer, 1991; Kirmayer, 2001; Nichter, 2010). In cases when these representations point to conceptualizations of disorders that differ markedly from Western diagnostic categories, the terms *culture-bound* and *cultural syndromes* (or culture-specific sets of symptoms reflecting a known form of distress; Lewis-Fernández et al., 2014; Hinton and Bui, 2019) are used, with a number of such syndromes and cultural etiologies (e.g., Cambodian *baksbat* or Andean or Mexican *susto*) suggesting relevance to trauma. More recently, the concept of *idioms of resilience* (or culturally shaped ways of understanding resilience) has been introduced in medical anthropology with the goal of understanding the full range of complex representations of subjective consequences of trauma (e.g., Lewis, 2013; Vindevogel et al., 2015). These approaches capture many of the complex ways in which people describe and explain causes and character of their suffering. Research using these concepts has generated much important work on trauma (e.g., Hinton and Lewis-Fernández, 2010; Molendijk et al., 2016; Lee et al., 2018), demonstrating that cultures differ when it comes to models of mental illness and linking clinical research to important scholarship on narratives and communication. This work has led to better understanding of the ways in which personal and shared meanings of distress differ across cultural groups (Maercker et al., 2019). Cultural and clinical (and cultural-clinical) psychology stands to benefit from this conceptual wealth.

Due to the very complexity of these models and their epistemological foundations, there are also some challenges to importing this set of concepts into psychology and generating empirically verifiable predictions. This paper aims to preserve some of their complexity while also harnessing the conceptual and empirical tools of cultural psychology to develop a concept of cultural scripts of trauma. The long-term goal here is to advance generative psychological research in this area. A reader may notice that our model of scripts resembles cultural syndromes, models of illness, or other existing models in some respects. A goal of introducing the new term of cultural scripts is to draw attention to the psychologically important characteristics of models of distress and point to ways of operationalizing and assessing them rather than to reimagine this domain.

In developing a model of cultural scripts, we aim to preserve and further develop a set of parameters that appear in the literature on models of trauma. These parameters are also important to the work on cultural shaping of psychological functioning in cultural psychology. First, it is important for us to consider the subjective (e.g., what I know), intersubjective (e.g., what I think others know), and culturally shared (e.g., what all of us actually know) knowledge about trauma and assess gaps between these representations (Chiu et al., 2010). Cultural models may reflect cultural consensus (culturally shared) and intersubjective cultural knowledge (Chiu et al., 2010; Fuchs, 2010). Because psychological functioning is responsive to both types of models (De Leersnyder et al., 2015; Schwarz et al., 2016), these ideas about trauma are important to understand and assess. Second, we need to maintain focus on the differences between ideal, normative, and pathological paths in responses to trauma (echoing the work on idioms of distress vs. resilience). The ways in which mental illness disrupts psychological functioning should be considered against cultural referents of what it means to be a normally or an ideally functioning person (Chentsova-Dutton and Ryder, 2019). Understanding how these paths are represented is especially important for traumatic stress, as traumatic events are commonly construed as turning points that divert people’s lives off previously imagined normative or ideal tracks (Davis and Lehman, 1995). Finally, it is important to understand dynamic features included in models of posttraumatic changes, echoing prior work on illness narratives or narratives of suffering (Zarowsky, 2004), and on past and future selves (e.g., Markus and Nurius, 1986).

## The Concept of Cultural Scripts

### Definition and Main Characteristics

Cultural scripts are types of cultural models that capture dynamic sequences of elements. This concept originates in broader cognitive psychology work on schema and artificial intelligence (Schank and Abelson, 1977; Abelson, 1981) and draws on the literature on the effects of schema on processing information (e.g., Mandler, 1984). Although this concept has been generative beyond cognitive psychology and has been previously adopted by scholars to describe cultural models of stress and distress (e.g., Long, 2004), much of the work that uses this term outside of psychology does not emphasize the specific empirical predictions for psychological effects of scripts. We aim to revisit some of these predictions here.

The script was defined by Schank and Abelson (1975) as a, “predetermined, stereotyped sequence of actions that define a well-known situation” (p. 151). A script is a *type of schema, one with a temporal organization of its elements*. It captures information about causal chains of phenomena that occur and reoccur in the same or similar sequence. It is common for script representations to have “tracks” (Den Uyl and Van Oostendorp, 1980), or representations of variations in sequences of actions or events depending on situation. Moreover, as with most mental models, scripts are typically encoded as families of related schema that inform each other. Most cultural scripts describe steps one can observe or personally undertake. As such, they are “in the head” as intersubjective norms of what happens (e.g., norms of behaving during a funeral), as well as “in the world” as observable and structured steps (e.g., burial rituals). The concept is similar to work in anthropology that captures dynamic cultural models as thoughts and emotions as well as ritualized behavior (e.g., Xygalatas, 2014). Although initially described as largely behavioral, scripts can also encode chronological sequences of rich psychological phenomena, including elements such as memories, attention patterns, bodily sensations, emotions, motivation, coping attempts, and ritualized behaviors.

The concept of scripts is well suited to describing how the sequential nature of posttraumatic changes is conceptualized in people’s minds. It shares certain similarities with process models of mental disorders in which a distinction is made between initial reactions, intermediate phases, and final states of disorders. Consider the first model of posttraumatic stress disorder (PTSD) proposed by Horowitz (1997). This model describes an affective outcry as an initial reaction, followed by primarily visual intrusions and conscious avoidance as a coping attempt, resulting in feelings of guilt and a consolidation of trauma-related memories. This sequence can be considered a script. What was missing in this—as it turned out—westernized script of PTSD was descriptions of bodily sensations, ritualized behaviors, and interpersonal elements that typify traumatized persons from other cultural groups (Summerfield, 2001).

Indeed, many, if not most, psychological scripts are shaped by culture. They require an understanding of “local cultural practices” (Searle, 1983, p. 144) or culturally shaped defaults of how things are or should be, describing steps one takes in culturally informed situations, ranging from the ordinary, such as routine social interactions (e.g., in Hispanic contexts: Triandis et al., 1984) to out-of-ordinary, such as mourning rituals (e.g., in Japan: Klass, 1996) or suicides (e.g., in US and Canada: Canetto, 2015). For example, a contemporary Chinese script for the first day of school involves predictable elements in a predictable order, including children going to their new classrooms, a joint singing of the national anthem, raising of the flag, the distribution of textbooks, often completed by a gathering that includes a speech by the schoolmaster and by students pitching in to clean the classrooms. Although the notion of starting a new school year on a good note is familiar to those outside of China, this specific script is not shared by cultural outsiders. It is linked to families of related Chinese scripts, such as those for a typical day at school, ways to celebrate or express patriotism, collective responsibility, and meeting new people or reconnecting to old friends. Culture can also influence the extent to which certain elements of the script or entire scripts can become “hypercognized” (Levy, 1973) or “hypersemiotized” (Hinton, 2012), due to their cultural salience and associations (e.g., the script of the emotion *omoiyari* in Japan: Lebra, 1976). Beyond tracks of the individual scripts, many cultural scripts are encoded as sets of alternatives, reflecting the existence of more than one way of navigating a particular challenge or responding to a given problem (e.g., Valentine’s, 2010 description of competing scripts of *grief* and *mourning* in Japan). People may shift between alternative scripts (e.g., Long, 2004) in their conceptualization.

As briefly mentioned, scripts are partly culturally shared and intersubjective in nature. They are understood as sequences that are not only familiar to oneself, but also to others, serving to ground interactions between people (Clark and Brennan, 1991). Building on the literature on intersubjective models in anthropology (Barth, 2002), scripts are conceptualized as guides for understanding and interpreting behavior as well as rules for behaving. Moreover, they need not be personally experienced to be understood and described by informants in a given cultural context. Consider the fact that most people in Western cultural contexts, including those with no personal experiences in this domain, are all-too-familiar with a cultural script of how premenstrual distress unfolds (Chrisler and Caplan, 2002). Many of the symptoms believed to be associated with this script have little validity, yet their endorsement is widely shared. This illustrates the fact that intersubjective scripts can be discrepant not only from the subjective beliefs and experience (e.g., “I personally do not experience or believe this, although others do”), but also from the culturally shared (e.g., average beliefs and behavior can be discrepant from the intersubjective norms, as with the college students’ perceptions of drinking norms: Baer et al., 1991). All three of these levels (subjective, culturally shared, intersubjective) are important to capture, as they can have different implications for psychological outcomes.

People are not passive recipients of their culture (Chiu and Cheng, 2010). Rather, endorsement and knowledge of cultural scripts and the extent to which individual symptoms align with these scripts differs person-to-person and subculture-to-subculture. Even the most inflexible of scripts generate a lot of within-group variance in how they are executed as well as in the attitudes about the script; these differences may appear against the background of relatively more homogeneous intersubjective representations. Imagine three Chinese people that have lost family members: one observes the traditional rituals (e.g., Kong, 2012), such as cleaning the grave site on the Tomb-Sweeping day and making offerings for the dead; another adopts Western practices and, although she visits the graveyard regularly, does not make the traditional offerings; yet another does not believe in souls and, although he grieves, rejects ritualized ways of mourning. Despite differences in what they believe and do, all three of these individuals may be able to report on the traditional Chinese mourning script with some fidelity. Of course, as ethnographic studies show, there may be specific environments and subcultures that limit familiarity with some scripts and increase familiarity with others (Brown and Closser, 2016). Some scripts are shared by most people in a given cultural context, while others may only be accessible to subgroups within a larger cultural context (e.g., Hmong healing practices in the USA; Plotnikoff et al., 2002). For example, a Chinese American living in China may not have the same understanding of traditional rituals as the locals, who may themselves disagree to some extent based on factors such as region or age.

For those familiar with it, the script would be important to keep in mind even if a given person does not confirm to it, as this person may need to explain their deviation from the shared script to themselves and others, with implications for how we understand their cultural competency (e.g., as performing or as knowing: Kleinman and Benson, 2006; Dressler et al., 2007). Failing to mourn in culturally prescribed ways differs for someone who is familiar with the script (e.g., a person acculturated to Chinese culture) relative to a cultural outsider (e.g., a foreigner). People tend to be less aware of what is actually culturally shared rather than intersubjective (in our example, how an average person in this Chinese community thinks about death and memorializes their loved ones). This is partly due to the fact that some scripts are hard to observe directly and partly to the fact that statistical regularities are hard to detect more generally. Yet, in some cases, discrepancies from the lived culturally shared norm can also matter and be associated with unfavorable outcomes (e.g., consider members of ethnic minorities in Belgium who experience emotions in ways that are statistically deviant from the culturally shared norms: Jasini et al., 2018). In sum, intersubjective scripts of mental illness (and the ways in which these scripts are manifested and endorsed on average) frame people’s understandings of their symptoms rather than rigidly prescribe them to respond in particular ways. Deviance from an intersubjective or culturally shared script of traumatic distress is just as important to capture and understand as adherence.

In some cases, a domain is scripted in some cultural contexts but is less scripted (or not scripted at all) in others. One example is the script of Indian *dhat*, or semen loss anxiety syndrome (Akhtar, 1988; Deb and Balhara, 2013). This term describes symptoms of sexual dysfunction, anxiety, depression, and somatic symptoms that are ascribed to loss of semen during sleep. These symptoms occur most often in young men from rural areas. Although primarily diagnosed in India and Sri Lanka, it has been observed elsewhere (Sumathipala et al., 2004). The phenomenology of this script reads as problematic across cultural contexts, yet it no longer taps into a dominant script in Europe and North America despite having been described as such in these contexts historically. To an Indian layperson in a rural area, a report of such changes would tap into the salient cultural script of *dhat*, pointing to additional symptoms (e.g., loss of appetite, lethargy, guilt, Deb and Balhara, 2013) and responses to these symptoms (e.g., seeking treatment from an Ayurvedic practitioner, Bottero, 1991). It may still sound like a known (although less familiar and coherent) problem to an urban Indian or an Indian immigrant living in Western cultural context. In contrast, in Europe and North America, this set of symptoms is unscripted. The lay public or even psychologists may consider other disorders and/or focus on factors such as sexual orientation or relationship difficulties instead. Although scripts of trauma-related changes are common, an open-minded researcher or a clinician should always consider the possibility that the domain of traumatic stress may be unscripted or only loosely scripted in some cultural contexts, whether in its entirely or in some aspects of the model.

In sum, the concept of scripts captures culturally shared ideas about mental illness that: (1) acknowledge that symptoms are represented as dynamic sequences rather than static sets; (2) exists as families of schematic sequences; (3) can be assessed as mental concepts and also as overt behaviors; (4) are subjective and culturally shared as well as intersubjective; (5) are represented as sets of alternatives rather than single models; (6) can affect psychological functioning and meaning-making regardless of script adherence; and (7) differ across cultures in terms of whether a particular domain of mental illness is scripted, unscripted or somewhere in-between, and also in the details of the available scripts. We will now consider in more detail how this concept can be applied to the domain of traumatic stress studies.

### Scripts of Trauma-Related Changes

Like other scripts, cultural scripts of trauma-related changes can be relatively simple in their structure when one examines the specific elements and their sequential flow, but relatively complex when one attempts to map broader conceptual structures, such as alternative scripts that are available in a cultural context, variations of the given script (i.e., script tracks), and associated conceptual models (i.e., script families). Let us start with the focal script itself. Cultural scripts of trauma sequelae typically include only a few symptoms that are widely believed to be associated with each other and are perceived to have a common cause in encounters with traumatic events. Unlike rigid social scripts (recall the first day at Chinese schools), scripts of trauma are more loosely structured, or “sketchy” (e.g., DeJong, 1977). They allow for some heterogeneity in the symptoms and how they unfold. Few core elements are invariant in their presence and order, with other elements represented probabilistically. Elements that are relatively invariant might include the presence of preconditions for encountering trauma, e.g., historical events or personal history of family violence, *karma* from prior lives (see Kohrt and Hruschka, 2010). Naturally, these scripts would include the traumatic event, defined by ICD-11 as of “extremely threatening or horrific nature,” (Maercker et al., 2013; World Health Organization, 2018) – or, more commonly, a series of events. Finally, cultural scripts of traumatic stress typically identify some of the key ways of reacting to these event(s), both in the short run and also later on. Some elements, such as trauma-related memories and nightmares, appear in scripts of traumatic distress across cultural contexts, highlighting a common core in patterns of human responses to intense stressors. Many of such culturally invariant and widely shared symptoms are recorded in the conventional clinical descriptions of PTSD in the slightly different versions that appear in the Diagnostic and Statistical Manual of Mental Disorders (DSM) and the International Classification of Disorders (ICD) (Stein et al., 2014). Other elements may show high levels of salience and consensus within a cultural context, but not across cultures, indicating cultural shaping of models of trauma (consider Hinton’s (2012) description of pain being hypersemiotized in some cultural contexts).

Let us now introduce some of the sources of complexity that arises from the fact that cultural models are usually not homogeneous and that they form networks of associations that take meaning from each other. As with other scripts, cultural scripts of traumatic stress are best considered and assessed not as self-contained models, but as sets of alternatives and their associated families of models (e.g., alternative scripts of posttraumatic stress and spirit possession in Uganda: Van Duijl et al., 2010). In many cultural contexts, more than one script may be available for understanding responses to trauma. People may be exposed to scripts originating from their own cultural context as well as to those that are imported as part of cultural exchange or targeted public health interventions. When asked about psychological problems linked to the genocide of 1994, Rwandan informers studied by Bolton (2001) referred to several concepts. Use of the label that was most closely associated with the Western-based concept of PTSD was higher among respondents exposed to community outreach campaigns, highlighting the possibility that these campaigns may have introduced or reinforced this concept and the script associated with it.

People may shift between scripts of trauma or even intentionally choose them, with consequences for causal models, symptom presentation, and treatment seeking. Each of the alternative scripts may be recruited in particular contexts to organize ideas about particular types of traumatic events or particular aspects of being affected by them or getting help. For example, in the late 1990s, the first author consulted a distressed Russian grandmother. Her granddaughter suffered a horrific extreme sports accident, nearly dying and witnessing another person die of injuries in front of her. The grandmother described not knowing what to expect, as she considered at least three possibilities: a model of stoically coping with difficulties derived from war-time experiences of her own parents; a model of coping with suffering and grief derived from church; and a Western-based notion of PTSD based on American movies. Each of these models pointed to different clusters of concerns, and, most importantly, to different paths to treatment and recovery. To understand this family’s experience, we would need to be aware of all of the scripts available to them, identifying cues that may activate them. In addition to families of distinct scripts of traumatic distress, each script may include multiple “script tracks” (Den Uyl and Van Oostendorp, 1980) or normative understandings of paths that may range from optimal or functional, non-optimal, but still understandable and non-pathological, and pathological and dysfunctional. A Russian grandmother was able to generate examples of tracks within the post-war script (e.g., some people traumatized by the war appeared to function normally, save for the nightmares; but others, albeit able to function, remained socially aloof; still others stayed distressed and drank heavily). A typical example of an optimal trauma script tack would be to speak of a short-term shaking-up of one’s own life, but then to focus on expectations of rapidly and successfully overcoming these short-term consequences (echoing the concept of idioms of resilience). A non-optimal track might describe predictions for taking longer to overcome the expected difficulties after trauma. In contrast, a pathological or dysfunctional script track can consist of very idiosyncratic or extreme variants that are still understandable. Memorable script violations may lay outside the tracks, in the realm of unscripted distress (e.g., a person who developed mania following a traumatic death of a loved one).

Beyond individual scripts and their tracks, other cultural models may be important to consider. No cultural model exists in isolation, taking on meanings as part of its associations with other models. Hinton and colleagues describe this as a process of understanding local trauma syndromes within the context of local ethnopsychology, ethnophysiology, and ethnospirituality (Hinton and Good, 2016; Hinton and Bui, 2019). It is helpful to consider the links between scripts of responses to trauma and families of other schematic representations, such as models of traumatic events (e.g., scripts of violent abuse), models of biological versus mental processes, other forms of altered mental states, and/or psychological and physical distress and dysfunction (e.g., scripts of spirit possession, loss and mourning, depression), and typical forms of treatment (e.g., scripts of spiritual counseling or herb healing). In addition, in many cultural contexts, scripts of individual trauma become embedded within larger protoscripts of historical trauma and cannot be fully understood without considering them as well (e.g., in American Indian communities: Evans-Campbell, 2008). In anthropology, scholars typically start with learning about a context in depth to understand local meaning structures. Understandings of trauma and its specific manifestations among Cambodian refugees is predicated on the ways in which members of this community think of phenomena like grief, spirit visitation, and physiological functioning (see Hinton et al., 2013; Hinton and Good, 2016). Scripts of posttraumatic responses in Uganda or Vietnam cannot be understood and described in depth without referencing scripts of spirit possession or ghost apparitions, respectively (Kwon, 2008; Van Duijl et al., 2010). In psychology, it is more common for researchers to focus on a particular aspect of psychological functioning in an understudied cultural context (e.g., emotions) without closely attending to others (e.g., religion, relationships). Even when the work focuses on a certain script, it can be very important to attend to the broader sets of local models when considering scripts of trauma-related changes, whether by making an effort to simply become aware of them or by formally examining and assessing those models that are thought to be highly relevant to understanding trauma.

Finally, scripts of trauma can be understood as scripted deviations from cultural models of normalcy. Cultures differ in their ideas of what is ideal or normative. For example, a traumatized individual’s network of relationships is best understood in reference to what is normal. Living alone can be a marker of problems in some cultural contexts and a normal way to live in others. Scripts of traumatic stress can be contrasted to the ideal or normative scripts of life not impacted by the traumatic aftermath, with the salient discrepancies becoming a potential source of information. A veteran with PTSD may find that his anger and hypervigilance impact his ability to connect to people, with social impairments (e.g., precarious friendship, inability to maintain a long-term romantic relationship) creating a salient discrepancy with subjective, intersubjective, or culturally shared ideas of desirable social functioning (e.g., expectations for stable and supportive friendships and marriage). The perceived differences between what is and what one would ideally want, or what one feels that one ought to do or have are known to be associated with depression and anxiety, respectively (Higgins, 1987). Because of this, it is important to assess how scripts of traumatic stress compare to culturally shaped ideas of successful life management.

In sum, culturally sensitive assessment of scripts of trauma-related changes would capture core elements of the script as well as complex conceptual structures that are co-activated when thinking about the traumatic events and their consequences. This process involves attention to: (1) the extent to which a domain is scripted; (2) if it is scripted, the key elements of the script and its script tracks (or distinct sets of predictions for possible course of events); and (3) alternative scripts of traumatic stress, and associated cultural models, with particular attention to scripts of ideal and normative functioning.

### Scripts From a Transdiagnostic Angle

Historically, posttraumatic stress was described in the clinical literature as a syndrome, a categorical rather than dimensional construct (Horowitz, 1997). This conceptualization is still reinforced by the ICD or the DSM diagnostic categories describing PTSD, Complex PTSD, and Acute Stress Disorder as discrete syndromes. Similarly, some of the culturally specific descriptions of traumatic stress are syndrome-like in nature (e.g., Cambodian *baksbat*: Chhim, 2013). One question that is important to consider is whether disorders related to trauma are best understood as discrete categories versus indications of broader confluent domains of distress. Psychological reactions to a traumatic event can manifest as a broad mix of negative emotionality (e.g., depression, anxiety) as well as severe memory and consciousness dysregulations (e.g., flashback tendencies, dissociation) and sleep-wake disorders (e.g., insomnia, parasomnia), to mention just a few possibilities. Arguments have been made for transdiagnostic conceptualizations of these patterns (Nolen-Hoeksema and Watkins, 2011).

The transdiagnostic nature of traumatic stress means that its symptoms do not naturally lend themselves to being understood as a discrete syndrome any more than human behavior lends itself to be understood through the lens of personality types. Kirmayer (1996) has observed that although dimensions of posttraumatic symptoms, such as somatization, dissociation, depression, and anxiety are observed across cultures, there is little evidence that local idioms of distress systematically map onto categories similar to PTSD across cultures. Ethnographic work shows that in some cultural contexts, such as Rwanda and Cambodia, scripts of trauma are not easily distinguishable from other scripts of emotional distress and dissociation (Kirmayer, 1996; Bolton, 2001; Fox, 2003). We would therefore expect that within the domains of internalizing distress and dissociation, different cultural scripts might carve out conceptual space differently, requiring a broad and systematic approach. In Western cultural contexts or those with Western influence, ICD- and DSM-derived categories aid people in the task of distinguishing PTSD from major depression or dissociative disorders. In other cultural contexts, the same conceptual domain may be carved up differently. We would expect to see cultural similarities at the level of broader families of scripts of mental illness and cultural differences at the level of specific boundaries between cultural scripts of responses to trauma versus those for related disorders. The take-home message for researchers and clinicians is that they need to: (1) remember that categories that are familiar to them may be culturally shaped; (2) frame their inquiries broadly; and (3) be prepared for the possibility that culturally specific scripts for traumatic stress may show a different categorical organization than the one to which they are accustomed.

#### Psychological Consequences of Script Activation

Scripts of trauma and posttraumatic changes are important not only because they help us understand what people know about trauma. They can have important psychological consequences. One key domain of interest is attention and memory. Cultural scripts of responses to trauma tend to structure both personal memories and cultural accounts of trauma and its consequences. Research on cognitive effects of scripts suggests that they frame interpretations of events and serve as “cognitive cuing structures” (Bellezza and Bower, 1982), activating expectations, guiding perception and inference, and shaping memories. This happens even among those with first-hand experiences (e.g., Nakamura et al., 1985), demonstrating that scripts compete with experience when it comes to shaping long-term representations. Studies examining recall of experienced sensations, such as physiological changes associated with emotions, and symptoms of concussions, premenstrual distress, and depression, convincingly show that people’s retrospective reports are co-shaped by their actual experiences and salient scripts (see Ferguson et al., 1999; Chentsova-Dutton et al., 2015).

Script-relevant information is processed and remembered as a package, meaning that elements that are missing are often filled in, even if erroneously (Bower et al., 1979), confirming or even emphasizing culturally salient aspects of scripts (see Imada and Yussen, 2012). Seemingly inconsequential information that is script incongruent may be overlooked and forgotten. Notable script interruptions (e.g., failure of a treatment to make an expected difference, as in Lemelson and Tucker, 2017) can aid recall and recognition in the short term by serving as “tags” for script packages. Still, script-consistent information is more influential in shaping longer-term memory (Smith and Graesser, 1981; Davidson et al., 2000). Notably, although shorter-term memories may not last, they are still culturally noteworthy, as they provide people with short-lived, but important, opportunities to transmit stories to others. Accounts with a small number of unexpected, bizarre or supernatural elements can become “viral” and propagate precisely due to their short-term memorability and high likelihood of transmission from person to person (Norenzayan et al., 2006). Inclusion of a small number of counterintuitive elements (not too many or too few) into an otherwise scripted story can thus aid longer-term cultural transmission. For example, accounts of combat-related PTSD shared by veterans with the first author second- or third-hand commonly included “viral” elements not typical of contemporary North American illness narratives, such as ghosts and supernatural powers (e.g., premonition, ability to control thoughts of others) granted to the victim by the experience. Taken together, this literature suggests that cultural scripts of traumatic stress shape memories of trauma.

Although the most widely studied effects of scripts involve memory, scripts may also shape other psychological processes. The mere introduction of basic schematic categories (of which scripts are a subset) changes subjective and neural representations of bodily sensations (Petersen et al., 2014). Scripts can also change one’s interpretation of one’s subjective experience. When a salient script is activated, some people interpret normal fluctuations in somatic sensations as symptoms of a scripted illness (Mittenberg et al., 1992). Finally, the literature on expectancy effects suggests that scripts can scaffold emergence of script-consistent symptoms (Kirsch, 1999; Schwarz et al., 2016). Although none of the prior studies examined posttraumatic distress directly, they suggest that cultural scripts of posttraumatic changes may shape clinical reports for some people. For example, people of Latino descent in the United States have been shown to be more vulnerable to PTSD following exposure to trauma than members of other ethnic groups; one potential explanation is that the cultural script of *ataque ne nervios* facilitates experience of symptoms consistent with PTSD (for a review, see Hinton and Lewis-Fernández, 2011). Hinton et al. (2007) also pointed out that catastrophic cognitions can be built into what are we are referring to as scripts of trauma reactions, with interruptions of these cognitions playing an important role in possible interventions.

When studying the psychological effects of scripts, it is particularly important to assess not just subjective scripts but also those that are culturally shared and intersubjective. Although both subjective and intersubjective beliefs can shape psychological functioning (e.g., Zou et al., 2009), the associations of subjective and behavioral outcomes with intersubjective or cultural beliefs are often stronger (Shteynberg et al., 2009), with Gelfand and Harrington (2015) proposing that motives to mitigate uncertainty and threat, manage interpersonal impressions, and establish power can increase the likelihood that cultural norms serve as behavioral guides. Given that exposure to traumatic events is associated with uncertainty, perceptions of threat, and low levels of power, we would expect intersubjective cultural beliefs about trauma to be particularly important for explaining individual-level symptoms and behavior, particularly in potentially evaluative contexts such as healthcare settings. In turn, adherence to objectively measured patterns of culturally shared beliefs and behaviors has its own psychological benefits described in the literature on cultural fit. We now turn to the discrepancies between these levels and the effects of cultural fit.

#### Subjective-Intersubjective-Culturally Shared Discrepancies and Cultural Fit

Regardless of the degree of similarity to shared and intersubjective scripts, the posttraumatic symptoms experienced by a given individual are likely to be interpreted in reference to these scripts. When considered at the subjective level, many scripts represent a blend of knowledge and belief (Abelson, 1981), combining information that is perceived to be factual with belief-based, affectively laden, and personally relevant material. One may store culturally shared knowledge about posttraumatic changes as factual information but also have doubts about some symptoms, or draw on episodic memories from one’s own life in completing the subjective script. At the intersubjective level, scripts are largely knowledge-based, comprising sets of imagined shared expectations for relevant symptoms and the ways in which they emerge and change over time. Shared emotions about particular types of traumatic events and their cultural or historical relevance may also be captured at this level (e.g., Jacobs, 2011; Paez and Liu, 2011). Finally, culturally shared scripts are mere statistical representation of typical subjective beliefs for a given population. Given that people do not have direct access to the beliefs of others or cognitive tools to accurately average across these beliefs, it is common to see shared intersubjective assumptions that do not reflect the actual consensus and are shaped by other sources (e.g., media).

One fruitful area of inquiry is to capture similarities and differences in these representations and cognitive and affective discrepancies between them (Wan and Chiu, 2013). What does a given person (1) know and feel about a particular type of posttraumatic response and (2) think others know and feel about the same domain? What do others actually know and feel, on average? Recent work on cultural fit and its psychological effects suggests that person-culture discrepancies have important clinical implications (Soto et al., 2013; Yoo and Miyamoto, 2018). It is psychologically beneficial to be similar to perceived and actual others in one’s cultural context and to have experiences that make sense culturally (Fulmer et al., 2010; Götz et al., 2018). People who experience culturally salient emotions in ways that are similar to statistically averaged others in their cultural context report psychological benefits above and beyond the effects of the emotions themselves (De Leersnyder et al., 2015). These associations have also been observed for values (Lu, 2006) and motivation (Higgins, 2005), with Higgins arguing that “value from fit” is a broad mechanism accounting for these psychological benefits.

Although these effects have not yet been demonstrated with posttraumatic symptoms, evidence is accumulating for other indicators of distress, such as negative emotions, neuroticism, and shyness (Miyamoto et al., 2013; Kitayama et al., 2018). Experiences of distress that match intersubjective and/or culturally shared cultural scripts of mental illness may have better outcomes relative to other, unscripted, forms of distress, due to factors such as lower stigma or improved ability to capitalize on established strategies for managing symptoms (Kirmayer et al., 1995). There may be other mechanisms, such as generation of meaning making and reappraisals, factors associated with broad psychological benefits (see Park, 2010), or benefits for communication or empathy. Extant research demonstrates benefits from fit when using indices of both intersubjective and culturally shared norms. We do not yet know how the two types of discrepancies differ in their effects. This area of research is sorely in need of studies, especially given proliferation of efforts to introduce novel scripts of PTSD to non-Western cultural contexts and the discrepancies introduced by such efforts.

In sum, a concept of cultural scripts of traumatic stress can be of use to researchers in cultural and clinical psychology and clinicians. We now will review case examples from the literature, considering culturally universal and culturally specific elements of potential scripts from different cultural contexts.

## Preliminary Examples of Traumatic Stress Cultural Scripts

The following section provides potential examples of cultural scripts of traumatic stress using existing narrative accounts. It focuses on the most important types of severe traumatic events and their aftermath (see Liu et al., 2017). None of these illustrations cover the full range of the definitional features of cultural scripts that were described earlier in this paper (e.g., some do not even include a sequential order of scripted elements). Nevertheless, it is important to briefly consider actual contexts of traumatization and think about the extent to which the concept of scripts may be useful in these contexts. We aimed to provide examples from different regions of the world to highlight the fact that the task of capturing cultural scripts is important for the understanding of traumatic stress in Western and non-Western contexts alike.

### Experience of Sexualized Violence

Sexualized violence^1^ can strike in childhood or adulthood, potentially yielding different scripts. This section focuses on the consequences of prolonged or repeated sexual assaults. The new diagnostic system, ICD-11, offers the possibility to diagnose consequences of such trauma as complex PTSD, in which the classical PTSD symptoms are accompanied by various disturbances of self-organization, including affective dysregulation, negative self-concept, and relationship disturbances (Reed et al., 2019). Because the domain of sexuality more generally and sexual violence in particular tends to be taboo, there are poignant descriptions of sufferers searching for relevant scripts and constructing ways of understanding a previously unscripted or under-scripted domain of experience, with clear references to normative scripts of sexuality and relationships.

Stadtmann et al. (2018) conducted case surveys on perceived symptoms and, over time, symptom management among survivors of sexualized violence, with reference to the concept of complex PTSD. Based on these surveys, an attempt was made to describe a typical traumatic stress script for this group (constructed from a clinician’s perspective). It is divided into several phases, with emotional ignorance of specific stressful symptoms (e.g., bodily sensations) followed by overcompensation (e.g., self-harming through scratching or substance abuse). The outwardly perceptible disorder and help-seeking are described as the result of a “paroxysm” phase in which clients report various images of physical breakdown as well as exhaustion. They describe this latter phase as one characterized by uncontrollable outbursts, sudden increases, or recurrence of many kinds of symptoms: thoughts, emotions, memories, sleep disturbances, bodily sensations, dissociation, and interpersonal clashes. The clients become ready for treatment in the next phase, which is characterized by their deep disappointment with themselves, and subsequent self-disclosure, prompting them to seek and accept professional help. The contents of self-disclosure focus on reconstructed description of their traumatic experiences, with their extreme humiliation and disgust described as the causal conditions for their suffering.

The Stadtmann study points to a peculiarity of these traumatic stress scripts from a middle European country. Because no script was available to these survivors to make sense of what was happening to them while it was happening or in the immediate aftermath, their stories were constructed after a time lag between the traumatic experiences (i.e., earlier history of abuse) and the formation of a script. In the meantime, these individuals suffered from unscripted distress and tried to cope with their daily life and appear well-functioning. Retrospectively, the patients tend to feel regret and anger due to not being able to express the real causes of their suffering. The script only became visible to the outside world and intersubjectively represented after their mental breakdowns. The costs of unscripted distress, including difficulties with explaining one’s suffering to oneself or others and seeking help, are highlighted by these narratives. Also salient is the way in which scripts of responses to sexualized violence reference other cultural scripts in the domain of self-construal, relationships, and optimal life course.

These patterns become even more apparent when child sexual abuse (CSA) is involved. CSA has been and still is a particularly silenced experience for various reasons, e.g., the affected children having no concept of healthy sexual boundaries and no adequate outlets of expression, or the ways in which cunning perpetrators intimidate their victims and cover their tracks. Many survivors of CSA have not experienced violent assaults, but the sexualized violence took place in the context of a terrible abuse of trust between an adult and an often emotionally needy child (Clancy, 2009). The immediate experience for the children is often characterized by extreme confusion about and ambiguity toward what happened to them in the course of sexual assaults. As young children are still learning cultural scripts for appropriate relationships and often lack exposure to discussions of sexual behavior, childhood experience of CSA is typically unscripted, and, therefore, perplexing. In the absence of a script, children are faced with the emotionally and cognitively difficult task of making sense of their experiences. Only years later, when the growing child or adolescent becomes capable of understanding the nature of normative sexual behaviors, a reconceptualization of memories and reattributions of their meaning takes place. A previously unscripted, but confusing and troubling, experience becomes scripted as abnormal and damaging. A trauma script develops that emphasizes the cognition of betrayal (often not only toward the perpetrator, but also toward the parents who were not able to protect their children). At the same time emotions of shame, self-blame, and disgust become dominant. In later stages, this mix of self-detrimental cognitions and emotions transforms into persistent problems with self-depreciation and interpersonal disturbances (Clancy, 2009). The majority of CSA survivors that have experienced this kind of *betrayal trauma* shun the term “trauma” when narrating their ongoing suffering, adopting other ways to describe their script (Freyd, 1994). Others who have suffered physical violence during the CSA more easily accept it (Finkelhor, 1981). The use of this idiom may reflect understudied differences in scripts recruited by these survivors, pointing to the possibility of families of scripts in this domain.

#### War-Related Violence

In his book, “The evil hours: a biography of post-traumatic stress disorder,” former US veteran Morris (2016) shares scripted stories of people living with ongoing traumatic stress, including himself. He describes what it meant to be a soldier (in the Iraq war in the early 2000s) and, on the one hand, to have to harm or even kill other people, and, on the other hand, to experience extreme suffering when witnessing death so close. He describes core PTSD symptoms like flashbacks, anxious suspicion, withdrawal, and chronic insomnia, but extends this list by introducing a dynamic analogy of transferring bad energies: “like a bullet, it enters the body, angry, and with a surplus of power, eager to transmit it to whatever flesh it finds, doing its work and then exiting, leaving a troubled body behind, dragging a comet’s tail of memory, hope, and innocence through the air, looking for another body to complicate” (Morris, 2016, p. 43). His description reads as a blend of subjective and intersubjective scripts as well as an idiom of distress, with its emphasis on communicating *via* such metaphors. Intersubjective script elements mentioned in his account start with the estrangement from other people and inferred beliefs of others about the veterans. He writes about the “moral vertigo” as an ongoing disorientation of core beliefs and values. According to him, US soldiers alternately feel like heroes, embedded in good comradeship, and like garbage, hating themselves for their past and projecting this nihilism to their future. The latter observation had been described as “moral injury,” and is understood PTSD as the core script of US veterans’ trauma sequelae (Litz et al., 2009).

Soviet veterans of the war in Afghanistan in the 1980s also describe a script of post-combat traumatic reactions. Our description is based on first-hand accounts of the war veterans as well as second-hand accounts collected post-deployment (Alexievich, 1990; Skliar, 2016). In some cases, particularly those based on early cases of soldiers returning from combat, veterans and their family members describe struggling with not knowing how to make sense of unscripted distress. They point to discrepancies between the observed symptoms and the widely shared historical script of post-war stoicism among Russian WWII veterans. Nearly all accounts allude to loss of positive emotions, particularly interpersonal ones, such as kindness and love. Furthermore, and similar to the testimonies of the US veterans, these accounts note a pervasive sense of distrust, alienation, and inability to connect to those who have not served. The interpersonal implications of these scripts (e.g., the ability to trust and be trusted by ingroup versus outgroup members) are likely to differ in the US and Russia, countries with different cultural scripts of normative social relationships (e.g., Markowitz, 1991; Realo and Allik, 1999).

Excessive use of alcohol to cope with these symptoms is mentioned in most of the Russian accounts, with veterans making use of a common and culturally sanctioned strategy for regulating emotions (Pesman, 1995; Popova et al., 2007). It is clear from the accounts that these changes are perceived to be disruptive but normative within the first year after returning from the battlefield, with expected improvements characterizing normal adjustment and continuing symptoms signaling “brokenness” and “moral injury” in the following years. These accounts signal different script tracks, highlighting cases of optimal adjustment, as well as those trajectories where intensity or course of distress exceeded shared expectations for the “unfortunate but normative” track and veered onto the “pathological” track.

US/American and Soviet/Russian veteran accounts reveal shared elements of the personal scripts and reflect some perceptions of the intersubjective scripts. The veterans’ personal accounts are notable for their focus on emotions, memories, coping attempts, and interpersonal conflicts; however, they do not commonly refer to bodily sensations, dissociative disintegration, or ritualized behaviors. The two cultural contexts, although distinct, do not seem to promote these types of expressions of traumatic stress. Note that whereas the US cultural script of long-term traumatic stress actively references the concept of “posttraumatic stress disorder” and is consistent with it, the Soviet/Russian script is very similar to it without the veterans using or even knowing this concept. The core of the two sets of subjective scripts highlights being broken and moral injury, with culturally patterned scripts for particularly troubling signs of brokenness, signaling deviation from culturally specific normative scripts.

#### Exposure to Organized Violence

Witnessing and surviving genocide is an undisputed collective traumatic experience. The Cambodian Khmer Rouge terror in the late 1970s killed around 2.5 million out of around 8 million people. Chhim (2013), a Cambodian eyewitness of the genocide and a psychiatrist, described an idiom of distress called *baksbat* that emerged in connection with this terror and the losses. This idiom describes trauma-related symptoms with some overlap with depressive, anxious, and dissociative symptoms, highlighting the possibility that this cultural context does not script these domains as separate. The idiom literally means “broken courage.” Just as combat veterans reference “brokenness,” this idiom evokes scripts of what it means to be whole. The descriptions of this form of distress are centered on a feeling that the soul has already left or will leave the sufferers’ bodies, accompanied by ongoing and extreme fearfulness. Descriptions of “soul loss” are also reported in trauma-related idioms of distress from other cultural contexts, such as *kesambet* in Indonesia (Wikan, 1989) or *susto* among Mexicans (Glazer et al., 2004). People with baksbat are characterized by exhaustion and passivity, and are less involved in life than may be expected. They suffer from physical symptoms such as headaches and digestive problems. Pale appearance and cold extremities are also common (Chhim, 2013, p. 166). Those affected by baksbat often think that their survival is only made possible by keeping their mouths shut about the horrible events they experienced and by being submissive. The baksbat script is an example of how the subjective script fits with the intersubjective expectations generated by a Buddhist principle that detachment from the outer world and oneself gradually relieves suffering^2^. It is also an example of a script that is far more inclusive of somatic sensations. Investigation of this script would be incomplete without understanding cultural scripts relevant to the intact soul and the ways in which it organizes normative or optimal functioning.

Another genocide took place in the 1990s in the African country of Rwanda. In 3 months, one million people were extinguished. The surviving refugees were displaced to camps, with a high additional death toll and trauma. Hagengimana, one of the only two Rwanda-based psychiatrists at that time, described *ihahamuka* (“lungs without breath”) as a common post-trauma “cultural syndrome” (Wulsin and Hagengimana, 1998; Hagengimana et al., 2003; Hagengimana and Hinton, 2009). This script largely mirrors a Western script for panic attack or panic disorder, and is usually triggered by trauma-related cues or flashbacks. The main focus of concern in this form of distress is acute bodily dysfunction, with somatic complaints in the form of frequent headache, gastrointestinal symptoms, dizziness, palpitations, and shortness of breath (Hagengimana et al., 2003). Hagengimana and Hinton (2009, see also Hinton and Hinton, 2014), argued that the culturally salient concepts of flow and blockage shaped description of the traumatic events and responses to them. During the genocide, the flow of people was stopped at roadblocks, with ensuing atrocities. In descriptions of *ihahamuka*, images of blockage of breath are emphasized, with restoration of flow central to scripts of healing. As part of experiencing *ihahamuka* attacks, people report fear of death. Once again, the elements of this cultural trauma-related script emphasize bodily sensations without describing the more emotional or cognitive script elements that make up the content of the PTSD definition in the ICD or DSM. One area for future research is to examine the ways in which this script activates ideas of culturally normative bodily functioning, as well as meanings attributed to these bodily complaints.

#### Political Oppression

Closely related to organized violence is systematic persecution of political opponents. In the 1990s, the second author examined former *political prisoners* of the German Democratic Republic; i.e., the portion of Germany that had been a separate communist country for 40 years. These prisoners had experienced various forms of physical and psychological abuse or torture (Maercker and Schützwohl, 1997). In retrospective reports, those who received a diagnosis of PTSD, made on average two decades after their release from prison, reported a scripted temporal sequence of signs and symptoms of traumatic stress. The immediate post-arrest period was dominated by emotions and cognitions related to fear and constant counterfactual rumination and/or self-blame over the reasons for their arrest. In the period after their release from prison (after an average 36 months in detainment, often in solitary confinement), cognitive schemata of “brokenness” dominated their clinical presentations, accompanied by negative self-evaluations of being permanently damaged or of “stolen life time”. Once again, we see clear reference to scripts of what it means to be unbroken or living a normative life in a particular cultural context.

Perhaps appealing to intersubjective perceptions, the former prisoners organized their self-presentation around their status as victims. Being socially acknowledged was far more important to them than financial compensation (Maercker and Müller, 2004). This particular Central European group of mainly males thus offers us an indication of a cultural trauma script that corresponds to the diagnostic definition of PTSD for the initial period after imprisonment, with shifting emphasis to subjective and intersubjective self-evaluative cognitions following this period. Hardly any of the respondents reported physical symptoms. Persistent dissociative symptoms were never reported spontaneously and were only acknowledged after specific prompts. In response, some reported a tendency toward drifting away and spacing out, continuing to the present day. Although the survivors attributed these experiences to their time in solitary confinement, this provides us with an example of symptoms that are not scripted, and, as a result, are experienced but not foregrounded in the survivors’ descriptions.

The examples presented here cover only few of the known scripts of traumatic stress. They showcase the transition from unscripted to scripted narratives of sexual traumatization and Soviet-era combat-related distress. All of them exemplify the “sketchy” nature of the established scripts, as only a few elements of these scripts are broadly shared or invariant across informants. Our summaries may make the scripts we outline appear to be inherently homogeneous. However, available data do not allow the readers to evaluate the heterogeneity of descriptions in each of the presented traumatic contexts. In examining these scripts, it is clear that phenomenological fields of traumatic stress include distressing and disruptive changes in the domains of intrusive symptoms and dissociation, emotional, somatic, cognitive and interpersonal dysfunction, as well as disturbances in self-construal, spiritual wellness, and “moral vertigo.” It is also clear that different clusters of symptoms are foregrounded as important in different cultural contexts, with other, unscripted, symptoms not acknowledged or acknowledged only after direct questioning. These summaries can be cautiously interpreted as indicating that cognitive and emotional changes are foregrounded in scripts from Western countries, while bodily sensations and spiritual concerns are foregrounded in scripts from non-Western countries.

One could ask whether we need to identify separate scripts for each trauma category (i.e., war-related, sexualized violence) versus focusing on commonalities. We know little about whether people tend to blend or differentiate these scripts. A primary argument against the idea of being too narrow is that, in addition to the psychiatric tradition, the clinical reality is that individuals with trauma-related disorders experience an average of 4.5 different traumatic events (Liu et al., 2017). This means that in practice, cases of posttraumatic distress caused by a single traumatic event are rare. Typical traumatized individuals around the world are those who have been traumatized over and over, by different types of traumatic events. Future work on cultural scripts of traumatic stress should therefore identify more systematic aspects of scripted representations.

The descriptions considered here are derived from the researchers’ and writers’ summaries of many individual’s stories of responses to traumatic events. Although these summaries reflect cultural scripts, they make it impossible to cleanly differentiate cultural scripts of what typically happens from individual experiences of what happened to a particular person. Future research on cultural scripts of traumatic stress will need to assess scripts as separate from individual experiences. In the next section, we will describe several methodological approaches to doing so.

## Research Methodologies for Assessing Cultural Scripts

Although clinical psychology has developed a rich array of tools for assessing the psychological impact of trauma, these tools tend to focus on the individual sufferer rather than the cultural context. Moreover, assessment is typically limited to affected individuals, such as those who directly experience symptoms of mental illness and/or their family members. The project of capturing cultural scripts of trauma in research and clinical practice would require cross-fertilization between clinical and counseling psychology and psychiatry and fields that study culture, including cultural psychology, anthropology, and sociology. A number of research approaches from these fields (for a review, see Cohen, 2019) are suitable for this goal. Given that intersubjective cultural scripts are available to many, at times most, people in a cultural context, many of the tools for assessing them aim to capture shared representations of community members, whether affected by mental illness or not.

### Ethnographies and Interview-Driven Approaches

When starting a research project in an understudied cultural context, an ethnographic approach that examines locals’ understanding of the domain of interest is recommended (Byrne, 2001; Reeves et al., 2008). Ethnographic data have been used to capture cultural models of mental illness across many contexts (e.g., Heurtin-Roberts et al., 1997; Lopez and Guarnaccia, 2000; see Manson, 1997). In-depth interviews with survivors or community members can be immensely helpful as a first step in understanding local experiences with and beliefs about traumatic stress, even if investigators aim to eventually progress to other methods of assessment. An ethnographic approach can be very valuable in introducing researchers and clinicians to families of scripts that are relevant to traumatic experiences in a given cultural context. Using ethnography can ensure that the researchers do not prematurely foreclose lines of questioning. Another advantage of the ethnographic approach, when done well, is that it encourages researchers to examine their own beliefs as much as those of the informants, directing attention to their own culturally shaped expectations. Mixed-methods approaches that combine strengths of the qualitative and quantitative traditions (Bartholomew and Brown, 2012) can also be an asset (see Jones and Kafetsios, 2005).

Once the scope of script-relevant domains in a given cultural context becomes better understood, it may be possible to create semi-structured and structured interviews assessing cultural scripts of trauma and posttraumatic responses. The Cultural Formulation Interview for the DSM-5 (CFI; Lewis-Fernández et al., 2014) provides an example of how to frame and structure questions for examining patients’ beliefs about their distress. A longer assessment tool that examines temporal beliefs is the McGill Illness Narrative Interview (MINI; Groleau et al., 2006). Although neither tool was created specifically to assess scripts of trauma, the CFI and the MINI include a number of questions that can be very helpful for this goal. As such, they can serve as the points of departure for developing interviews to assess scripts of trauma.

To fully assess relevant script(s) of trauma, a researcher would need to include questions focusing on local understandings of: (1) traumatic events, including whether they are understood to be different from other significant stressors, such as bereavement; (2) factors that predispose someone to experience traumatic events or be vulnerable to their effects (see Kohrt and Hruschka, 2010); (3) immediate, short-term, and long-term responses to trauma, with attention to all relevant domains of responding, including somatic, affective, motivational, cognitive, dissociative, interpersonal, and spiritual, as well as any additional symptom domains that are suspected to be relevant in a given cultural context based on ethnographic data; (4) different tracks or trajectories of responses, including informants’ understanding of the best-case (optimal course), typical (normative course), and worst-case (pathological course) scenarios for unfolding symptoms of distress and their duration. Finally, interviews might also include questions about; and (5) treatment options and effectiveness, including all locally available options, such as self-treatment, official medicine, and local healing practices. A more clinical variant of these questions are the well-known “Kleinman questions” in medical anthropology (Kleinman, 1980). Another, more systematic assessment method is the Event Chapter Sorting Tasks (with participants describing different chapters of responses to trauma, e.g., Dalgleish et al., 2011) that can facilitate structuring of complex temporal reports.

Another important dimension of beliefs about trauma can be added to interview or self-report assessments by explicitly asking participants to report on their personal opinions, beliefs and knowledge (subjective assessment) as well as those reflecting perspectives of their community members (intersubjective assessment). Any question can be framed to yield both types of responses (e.g., What do you personally believe are the best ways to treat these symptoms? What would members of your community say?). See, for example, the way in which the questions are framed in the Cultural Formulation Interview. Culturally shared beliefs can be estimated by averaging over subjective reports. When culturally shared and intersubjective beliefs are captured, researchers and clinicians can use the information to assess and address subjective-intersubjective and subjective-culturally shared discrepancies and adaptations to them. Although assessment of subjective and intersubjective beliefs side-by-side requires time and effort, there is much to be gained from incorporating both (as well as culturally shared beliefs that are captured by assessing the statistically shared subjective beliefs) into the methodological repertoire of clinical psychologists.

### Cultural Products

Another approach from cultural psychology is assessment of cultural models portrayed in publicly available and widely shared cultural products, such as songs, books, religious texts, newspaper stories, greeting cards, and paintings (Morling and Lamoreaux, 2008; Lamoreaux and Morling, 2012). Cultural products reflect and reinforce salient cultural dimensions, such as power distance (Lamoreaux and Morling, 2012), as well as more specific culturally shaped models of psychological processes, such as emotions and motivation. Consider, for example, a study by Koopmann-Holm and Tsai (2014) that compared German and US sympathy cards given to those who have lost someone. The German cards were more likely to acknowledge grief and portray images of death and less likely to offer positive acknowledgements and portray living images than the US cards. These differences were consistent with self-report data showing that people in Germany avoided negative affect associated with grief and suffering less than those in the US.

Cultural products portraying post-trauma outcomes may include songs (e.g., war songs), stories, books, newspaper articles, and online discussions. Even children’s books may be used (e.g., *Bridge to Terabithia*; Paterson, 1977). For systematic analyses, researchers select representative samples of cultural products that describe trauma or mental illness (e.g., all fiction describing trauma published within a certain time period) based on random selection or popularity. Trained researchers code each cultural product. For example, type of event, descriptions of preconditions for trauma, or traumatic stress, initial and longer-term reactions may be coded, with attention to indicators of whether the description depicts normative or pathological outcomes. The use of this approach is constrained by sheer availability of cultural products that openly discuss trauma and by the type of material that is included in such accounts. As such, this tool may be better suited for studying trauma in some cultural contexts than in others.

### Situation Sampling

Due to the fact that scripts of trauma are often organized around traumatic events, situation sampling is another tool from cultural psychology that can be valuable (Kitayama et al., 1997). This approach is rooted in the assumption that many cultural differences are driven by situational affordances and/or by their public meanings. Situation sampling has been used to study a range of psychological phenomena, such as social support, honor, and emotions (Uskul et al., 2012; Boiger et al., 2013; Morling et al., 2015). A two-step approach allows researchers to disambiguate the impact of cultural differences in occurrence of particular types of situations (e.g., types of traumatic events, such as traffic accidents, episodes of sexual and physical violence, natural disasters) from the impact of cultural scripts that guide responses to them. This approach acknowledges the fact that traumatic events happen with different frequencies in different cultural contexts and may have different characteristics, potentially triggering different types of symptoms and ideas about them. Some of the observed differences in cultural models of trauma may be driven by the prevalence of particular types of traumatic events and by the situational features that characterize them (e.g., response of other people, available resources).

In the first phase of a situation sampling study, researchers ask people from several cultural contexts to describe their experiences of typical or recent events with particular characteristics. To study trauma, one might ask people to describe traumatic events experienced by them or by someone in their social network. In addition to event descriptions, one would also obtain ratings for these events on dimensions such as typicality, controllability, or emotional response to the events, and/or code obtained descriptions for the typical characteristics of the events described by participants in each cultural context. This step allows one to better understand what types of traumatic events are typically encountered in each cultural context.

In the second stage of a situation sampling study, researchers select a subset of events from those generated during the first stage, whether by randomly drawing descriptions from each set or by selecting events that are described most frequently or rated as most typical, forming sets of events from each of the cultural contexts under study. They are then translated and back-translated, with use of cultural consultants and cognitive debriefing (testing it with intended participants, carefully interviewing them about their understanding of the questions and response strategies), if necessary (see Wild et al., 2005 for best practices recommendations). Let us imagine that one is studying trauma in Romania, Germany, and France. After completing step 1 of the situation sampling study, one would gather sets of traumatic event descriptions from each of these contexts, translating and back-translating them and creating a set of events containing equal numbers of Romanian, German, and French events, translated into the three languages. New samples of participants from each cultural context would rate these descriptions on scales of interest (e.g., typicality, controllability) and provide a hypothetical set of normative, worst-scenario, and best-scenario responses that might be expected for those encountering these events and adjusting to them in the short- and long-term. The resulting data allows one to examine the impact of the original source of descriptions (e.g., do the Romanian events engender higher expectations of anxiety relative to the German and French events?) as well as the impact of the participants’ mindsets (e.g., do Romanians report higher expectations of disassociation relative to Germans and the French?). The interactions between these factors can also be examined to determine whether events from one’s own context engender different responses than events from other contexts. To our knowledge, this time-intensive but versatile research tool has not been used by clinical psychologists, yet it offers promise as a means of characterizing cultural scripts of traumatic stress.

#### Consensus Analysis

In cognitive and developmental work on scripts, consensual models are established by simply asking people to generate familiar scripts and documenting which of the elements are endorsed by the majority (Bozinoff, 1982). Consensus analysis provides a more sophisticated tool for assessing consensus (Romney et al., 1986; Caulkins, 2004). This approach is well suited to examining cultural models of disease (Lynch and Medin, 2006; Dressler, 2012; Ulijaszek, 2013). Consensus analysis originates in anthropology and has been increasingly adopted by cultural psychologists (e.g., Boiger et al., 2014). It allows researchers to examine whether evidence supports a consensual cognitive model in a given domain (e.g., symptoms that are understood to be characteristic of a particular illness). Although very few studies have used consensus analysis to examine beliefs about traumatic stress (e.g., Alemi et al., 2017), the approach has been used to study related forms of internalizing distress, such as depression, *susto*, and *nervios* (Baer et al., 2003; Lynch and Medin, 2006). It has also been applied to clinicians’ understandings of the taxonomy of mental illness (Flanagan and Blashfield, 2007).

Researchers performing consensus analysis start by identifying homogeneous sets of questions (e.g., symptoms). As such, consensus analysis always comes in the wake of ethnographic and/or self-report studies, with prior data informing researchers about the sort of information that falls in a given domain in a given cultural context. In the domain of trauma, this approach can be used to examine relatively focused sets of questions, such as “is there a consensual model of typical bodily sensations/spiritual symptoms that are associated with trauma?” Informants in a consensus analysis study are provided a resulting set of questions and asked to rate these questions’ relevance or significance to the domain.

The resulting dataset is then factor analyzed, with participants serving as variables and their responses as cases. This analytical approach requires small samples, with the number of participants commensurate to the number of questions. A key assumption here is that shared consensual models are so accessible that they can be detected using relatively few informants; however, some researchers have opted for larger groups of participants, randomly sampling smaller subsamples to verify consensus (Segalowitz et al., 2016). Data that indicate the presence of a single factor in participants’ responses are interpreted as evidence of cultural consensus, with the presence of more than one factor interpreted as indicating cultural complexity in the representation of a given domain (Lacy et al., 2018). A useful feature of this approach is that it provides individual participants’ loadings onto a consensual factor (or their “competence scores”), as well as allowing researchers to examine question difficulty (or their “cultural salience”). Some people may load highly, indicating that they are accurate carriers of consensual knowledge in a given domain; others may have weaker loading, indicating that their understanding of a given domain does not match the consensus. In clinical research and practice, it can be very helpful to know whether or not someone is a good carrier of a consensual model. The approach may be particularly useful for examining models of distress among immigrants, comparing their consensual loadings with those from the heritage versus local majority cultural contexts. In addition, this approach can be fruitfully used to examine within-culture differences based on factors such as timing of assessment (Dressler et al., 2015) and professional status (e.g., Lynch and Medin, 2006). Recent studies also compare consensual models across cultural groups (e.g., Weller et al., 2002; Anders et al., 2018), with multicultural extension methods allowing for formal cross-group comparisons.

In sum, methodological tools used to study culture can enrich the toolkit of clinical researchers. These tools include ethnographic and interview-driven approaches, studies of cultural products, situation sampling, and cultural consensus analysis. These approaches can complement existing ways of assessing traumatic stress, taking the field further.

## Conclusion

We have offered our readers a vision for integrating clinical and cultural approaches to traumatic stress, with a goal of fostering generative work in psychology. Although the field of culture and traumatic stress has developed a rich array of theoretical models and assessment tools, the addition of a cultural script approach would offer a way to capture complex and sequentially arranged models of traumatic stress that are subjectively understood and intersubjectively shared, pointing a way toward operationalizing and assessing complex cultural models of posttraumatic changes. This approach would allow clinical psychologists to better differentiate between scripted and unscripted forms of individual distress and attend to the possibility of alternative scripts. The cultural script approach to trauma encourages researchers and clinicians to consider psychological and social consequences of “scriptedness” and offers specific predictions for the impact of script activation on attending, remembering, experiencing, and communicating posttraumatic distress. Introduction of this concept can also help clinicians better understand and assess potential discrepancies between how one experiences distress and what is understood to be normative. Finally, given research on how scripted information propagates from person to person (for a review, see Chentsova-Dutton and Heath, 2009), understanding of scripts of traumatic stress can also help researchers model ways in which information about distress transmits within and across communities, with implications for health education efforts. In sum, this approach aims to integrate some of the most significant insights from the anthropology, transcultural psychiatry, cultural psychology, and cultural evolution literatures (e.g., Ross, 1999) with knowledge about trauma and its psychological consequences generated by psychiatry and clinical psychology.

Case studies of traumatic stress illustrate that its manifestations vary across cultural contexts. In the absence of scripts, survivors describe confusion and searching for explanations, as is the case for CSA survivors. When scripts are available in a cultural context, they serve as consensual frameworks for managing communication about symptoms and addressing them in culturally specific ways. The case studies highlight the fact that cultural scripts of traumatic stress do not just exist as depictions of distress. Rather, they shape symptoms by directing attention to and encouraging concerns about some aspects of phenomenological fields of responses to trauma and not others, thereby fostering culturally specific forms of suffering. The fact that some survivors of trauma, such as Cambodian genocide survivors, experience their distress as painful bodily symptoms, while others, such as former East German prisoners, report changes in emotions and sense of self suggests that cultural scripts of trauma manifest in experienced and reported symptoms, at least for some sufferers. As knowledge of these symptoms becomes transmitted from person to person, scripts of what might happen to someone post-trauma become widely shared.

Given cultural heterogeneity in clinical presentations and outcomes, a one-fits-all approach to assessment is bound to miss some culturally and clinically important information. An increased focus on cultural scripts needs to be accompanied by the development of better techniques to assess scripts and examine their psychological impacts in research and clinical settings. We have introduced a short and non-exhaustive set of exciting methodological approaches to studying cultural scripts in hopes that they may prove useful to the next generation of traumatic stress researchers.

In conclusion, the theoretical and methodological tools described here aim to improve the ability of researchers and practitioners in psychology to deliver effective care to those suffering from traumatic stress. They point toward the important dual projects of (1) characterizing families of scripts of traumatic stress and (2) understanding their clinical impacts and the subjective-intersubjective discrepancies generated by them. We have argued that cultural scripts aid survivors of trauma and care providers in making sense of the complex and potentially confusing phenomenological fields of potential symptoms. The concept of cultural scripts can similarly draw on the rich work on cultural models of distress and aid clinical psychologists in their efforts to develop parsimonious yet contextually sensitive models of traumatic stress.

## Author Contributions

YC-D and AM were involved in conceptualizing, drafting, and finalizing the paper. AM initiated the project and coordinated all stages.

### Conflict of Interest

The authors declare that the research was conducted in the absence of any commercial or financial relationships that could be construed as a potential conflict of interest.

## Footnotes

^1^The authors prefer the term *sexualized violence* over *sexual violence* in accordance with some international advocates’ statements that sexual activities are not central to this, but rather that it is about sexuality in power imbalance, with the exercise of dominance playing an essential role.

^2^Recently, the *baksbat* idiom of distress has been critically evaluated (Kidron and Kirmayer, 2019). The authors center their critique: first on the reification of a pathologizing category instead of using the concept as a more fluid description of a broad and flexible range of changes and coping attempts and second on highlighting the fact that *baksbat* is part of a more complex network of post-conflict societal or social psychological attitude changes.
